# Acute pancreatitis in pregnancy following COVID-19 vaccine: a case report

**DOI:** 10.1186/s13256-022-03607-0

**Published:** 2022-09-29

**Authors:** Rajib Kumar Dey, Hemamala Ilango, Subash Bhatta, Ahmed Shaheed, Shanaz Dole, Ahmed Zooshan, Mohamed Faisham, Moosa Murad

**Affiliations:** 1 Department of Internal medicine, Indira Gandhi Memorial Hospital, Male’, Maldives; 2 Department of Gastroenterology, Indira Gandhi Memorial Hospital, Male’, Maldives; 3 Department of Otolaryngology, Indira Gandhi Memorial Hospital, Male’, Maldives; 4 Department of Obstetrics and Gynaecology, Indira Gandhi Memorial Hospital, Male’, Maldives

**Keywords:** Pfizer-BioNTech (BNT162b2) mRNA vaccine, COVID-19, Pregnancy, Pancreatitis

## Abstract

**Background:**

Since the approval of the Pfizer–BioNTech (BNT162b2) mRNA vaccine for COVID-19 infection, a few adverse effects have been reported. Acute pancreatitis has been reported in a few patients. However, there is currently no research showing a direct relationship between the vaccine and acute pancreatitis. Here, we report a case of acute pancreatitis following Pfizer vaccination in a young healthy pregnant woman without any known risk factors. To our knowledge, this is the first case report of possible vaccine-induced pancreatitis in a pregnant woman.

**Case presentation:**

The patient, a 24-year-old South-Asian female, at 31 weeks of gestation, presented with severe epigastric pain radiating to the back and worsening on lying supine, associated with nausea and vomiting. She was diagnosed with acute pancreatitis with a serum lipase level of 4376 U/L and an ultrasound showing features of pancreatitis. The patient received her first dose of the Pfizer vaccine 1 week prior to these symptoms. Detailed evaluation did not show any etiological cause of pancreatitis. The patient had a spontaneous vaginal delivery and the baby was shifted to the neonatal intensive care unit in a stable condition. A computed tomography scan postpartum (day 2) demonstrated acute interstitial edematous pancreatitis. The patient was managed conservatively in the intensive care unit and discharged home in a stable condition.

**Conclusion:**

This report highlights the importance of a detailed history and evaluation, and the close monitoring of any patient presenting with abdominal pain after vaccination. Acute pancreatitis can be fatal if not picked up early.

## Introduction

The Pfizer-BioNTech (BNT162b2) mRNA vaccine (Pfizer vaccine) against COVID-19 was granted emergency use authorization (EUA) by the Food and Drug Administration (FDA) in December 2020 [1]. There is very limited data in the literature about the adverse effects of the Pfizer vaccination [2, 3]. The association of acute pancreatitis with this vaccination has been mentioned in a few case reports [4, 5]. However, there is no research showing the direct relationship between the Pfizer vaccination and pancreatitis. The present study reports a case of acute pancreatitis following the Pfizer vaccination in a young pregnant lady without any known risk factors for pancreatitis. This is the first case report of its kind in the literature.

## Case presentation

The 24-year-old South-Asian female, primigravida, presented at 31 weeks of gestation with epigastric pain for 6 hours. The pain was sharp, radiating to the back and was worse on lying supine. Pain was associated with fever, nausea, and vomiting for the same duration. The patient denied any history of recent infection, or of consuming any new medication or herbal drugs. The patient was only on iron and folic acid supplements during the course of her pregnancy. The patient was a nonsmoker and had never consumed alcohol. Her past medical, surgical, and family history were not significant. The patient was a housewife and comes from a middle-class family. Her obstetric follow-up was uneventful so far. The patient received the first dose of the Pfizer vaccine 1 week prior to the onset of symptoms.

On general examination, the patient was conscious, oriented, and alert. Heart rate of 106 beats per minute, blood pressure of 140/90 mmHg, body temperature of 101°F, respiratory rate of 22 breaths per minute, and oxygen saturation of 98% in room air were recorded. On abdominal examination, no obvious skin rashes were visualized in the abdomen. There was abdominal distension with gravid uterus and severe epigastric tenderness. The cardiovascular, respiratory, and neurological examinations were unremarkable.

**Fig. 1:**
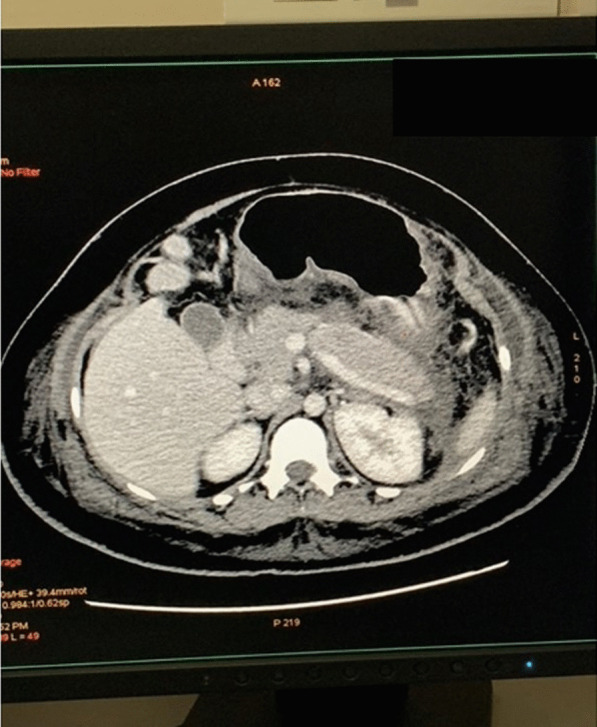
Computed tomography abdomen revealing bulky pancreas with mild enhancement, marked peripancreatic fat stranding with inflammation. (patient’s details shadowed)

The initial blood investigations revealed a total leukocyte count of 17 × 10^9^/L, serum lipase of 4376 U/L, and serum amylase of 83 U/L. An ultrasound scan of the abdomen showed features of pancreatitis. Her gall bladder was normal and no gallstones were seen. Acute pancreatitis was diagnosed and treated with intravenous hydration, antibiotics (intravenous ceftriaxone 1 g twice daily for 7 days), proton pump inhibitors (intravenous pantoprazole 40 mg twice daily for 5 days), and pain relief (intravenous paracetamol 1 g every 6 hours for 3 days) in the intensive care unit (ICU). A real-time reverse-transcriptase polymerase-chain-reaction (rRT-PCR) for SARS-CoV-2 screening was done prior to admission and was negative. On the second day of admission, the patient had a spontaneous vaginal delivery and the baby was shifted to the neonatal ICU in a stable condition. In view of ongoing upper abdomen pain, a computed tomography (CT) scan of the abdomen was performed, which revealed bulky pancreas with mild enhancement and marked peripancreatic fat stranding with inflammation, suggestive of acute interstitial edematous pancreatitis (Fig. 1). Over the next 48 hours, her pain settled and slowly oral intake was encouraged. Blood cultures taken at admission were sterile. The pancreatic enzymes on day 4 of admission were in the normal range. Her diet was changed to regular after the improvement of symptoms, and the patient was discharged in a stable condition on day 7. She was reviewed at 1 month and again after 6 months in the clinic and has remained stable since then.

Detailed evaluation of all possible etiologies for pancreatitis did not reveal any specific etiology. Her liver function tests, calcium, and triglycerides were normal since admission (Table1). Her antinuclear antibody(ANA) was negative. Her ultrasound done at admission and a magnetic resonance cholangiopancreatography (MRCP) at 3 weeks did not show any gallstones. Her pancreatic duct was normal and there was no abnormal pancreaticobiliary union.

**Table 1:** Laboratory parameters during admission and follow-up

Laboratory parameters	Day 1	Day 4	Day 30
Hemoglobin, g/dL	15	14.2	14.6
Hematocrit, %	46	45	45.2
Leukocytes, 10^3^ mL	17	10.6	9.4
Neutrophils, %	85	72	72
Lymphocytes, %	9	23	22
Platelets, 10^3^ mL	171	220	264
Blood urea nitrogen, mg/dL	8	10	10
Serum creatinine, mg/dL	0.54	0.7	0.8
Serum sodium, mmol/L	135	137	
Serum potassium, mmol/L	3.8	3.7	
Serum calcium, mg/dL	8.5	8.7	
Serum phosphate, mg/dL	2.8		
Serum magnesium, mg/dL	1.9		
C-reactive protein, mg/mL	4.64	1.02	0.5
LDH, U/L	236	210	196
Total bilirubin, mg/dL	0.9	0.9	0.9
Direct bilirubin, mg/dL	0.3	0.3	0.3
ALT, U/L	9	11	15
AST, U/L	21	24	22
ALP, U/L	112	120	108
GGT, U/L	12	14	16
Serum protein, g/dL	6.7	6.9	7
Serum albumin, g/dL	3.5	3.7	3.8
Serum amylase, U/L	836	76	62
Serum lipase, U/L	4376	68	40
Total cholesterol, mg/dL	188		
Serum triglycerides, mg/dL	140		
LDL cholesterol, mg/dL	132		
ANA	Negative		
rRT-PCR SARS-CoV-2	Negative		
HBsAg	Negative		
Anti HCV	Negative		
HIV antibodies	Negative		
Urine routine	No proteins. No pus cells/RBCs		

*LDH* lactate dehydrogenase, *ALT* alanine transaminase, *AST* aspartate transaminase, *ALP* alkaline phosphatase, *GGT* gamma-glutamyl transpeptidase, *LDL* low density lipoprotein, *ANA* antinuclear antibody, *rRT-PCR* real-time reverse transcriptase polymerase chain reaction, *SARS-CoV-2* severe acute respiratory syndrome coronavirus 2, *HBsAg* hepatitis B surface antigen, *Anti-HCV* anti-hepatitis C antibody, *HIV* human immunodeficiency virus

## Discussion

Acute pancreatitis has been linked to various vaccines in the past including inactivated influenza, Mumps–Measles–Rubella (MMR), and Human Papilloma virus [6–8]. A few cases of acute pancreatitis have also been reported after the Pfizer vaccine. Prakash *et al.* [4] reported a case of acute pancreatitis in a 96-year-old patient that developed a few days after administering the first dose of the Pfizer vaccine. Cieślewicz *et al.* [5] published a report of acute pancreatitis in a healthy 29-year-old healthcare worker who developed symptoms 20 hours after getting the first dose Pfizer vaccine. Acute pancreatitis has also been reported in SARS-CoV-2 infection itself [9].

In this study, the patient did not have any known risk factors for pancreatitis. The development of symptoms 7 days after the vaccination also pointed towards the Pfizer vaccine being the most likely cause behind acute pancreatitis. The mechanism of vaccine-related pancreatic injury is still unclear. Bogdanos *et al.* [10] proposed the molecular mimicry theory, which states that amino acid sequence similarities between viral and self-antigens can result in an autoimmune reaction. Vojdani *et al.* [11] found that autoantibodies against SARS-CoV-2 spike protein and nucleoprotein show cross-reactivity against many human tissue antigens. Using the Naranjo scale, pancreatitis caused by the Pfizer vaccine in this patient scored 5 “probable” [12]. The pregnancy was not assumed to be the cause of pancreatitis as an association between pregnancy and pancreatitis is very rare [13]. Eddy *et al.* [14] found that acute pancreatitis occurs in approximately 3 out of 10,000 pregnancies, while Sun *et al.* [15] demonstrated an increase in the incidence of preterm delivery in pregnant women with pancreatitis, as was seen in the present case report.

This indicates the need for close monitoring of patients presenting with abdominal pain after vaccination. It is difficult to conclude that the vaccine would have caused pancreatitis until we have data on long-term side effects. This is a new vaccine and long-term studies are required to establish a link between these vaccines and their potential side effects. Our purpose of writing this case report is to make all healthcare workers aware of the possible adverse effects of vaccines and to prevent any unfortunate events.

## Conclusion

This is the first reported case of acute pancreatitis following the Pfizer vaccination in a healthy young pregnant woman without any known risk factors. This study highlights the importance of close monitoring of any patient presenting with abdominal pain after vaccination. The recommendation has also been put forward for the need of a large-scale study to establish the association between vaccines and the emergence of adverse effects.

## Data Availability

Not applicable
